# *Escherichia coli* Type III Secretion System 2 ATPase EivC Is Involved in the Motility and Virulence of Avian Pathogenic *Escherichia coli*

**DOI:** 10.3389/fmicb.2016.01387

**Published:** 2016-08-31

**Authors:** Shaohui Wang, Xin Liu, Xuan Xu, Denghui Yang, Dong Wang, Xiangan Han, Yonghong Shi, Mingxing Tian, Chan Ding, Daxin Peng, Shengqing Yu

**Affiliations:** ^1^Shanghai Veterinary Research Institute – Chinese Academy of Agricultural SciencesShanghai, China; ^2^College of Veterinary Medicine, Yangzhou UniversityYangzhou, China

**Keywords:** avian pathogenic *E. coli*, ATPase, EivC, motility, virulence

## Abstract

Type III secretion systems (T3SSs) are crucial for bacterial infections because they deliver effector proteins into host cells. The *Escherichia coli* type III secretion system 2 (ETT2) is present in the majority of *E. coli* strains, and although it is degenerate, ETT2 regulates bacterial virulence. An ATPase is essential for T3SS secretion, but the function of the ETT2 ATPase has not been demonstrated. Here, we show that EivC is homologous to the β subunit of F0F1 ATPases and it possesses ATPase activity. To investigate the effects of ETT2 ATPase EivC on the phenotype and virulence of avian pathogenic *Escherichia coli* (APEC), *eivC* mutant and complemented strains were constructed and characterized. Inactivation of *eivC* led to impaired flagella production and augmented fimbriae on the bacterial surface, and, consequently, reduced bacterial motility. In addition, the *eivC* mutant strain exhibited attenuated virulence in ducks, diminished serum resistance, reduced survival in macrophage cells and in ducks, upregulated fimbrial gene expression, and downregulated flagellar and virulence gene expression. The expression of the inflammatory cytokines interleukin (IL)-1β and IL-8 were increased in HD-11 macrophages infected with the *eivC* mutant strain, compared with the wild-type strain. These virulence-related phenotypes were restored by genetic complementation. These findings demonstrate that ETT2 ATPase EivC is involved in the motility and pathogenicity of APEC.

## Introduction

A number of Gram-negative pathogens, such as *Escherichia coli* (*E. coli*), *Salmonella*, *Yersinia*, and *Pseudomonas* utilize type III secretion systems (T3SSs) to deliver effector proteins into eukaryotic host cells and to assemble flagella, which facilitate infections and bacterial motility (Mota and Cornelis, 2005; Diepold and Armitage, 2015). The structure of the T3SS apparatus, known as an injectisome, is evolutionarily conserved among pathogenic T3SSs and flagella, which share a similar membrane-spanning basal body. T3SSs have an extracellular, needle-like projection connecting to the basal body, which serves as a channel for the translocation of effector proteins. These bacterial effector proteins interact and subvert the innate immunity of host cells, thereby facilitating bacterial growth and survival, which subsequently lead to diseases (Cornelis and Van Gijsegem, 2000; Coburn et al., 2007). In contrast, the basal body of flagella, which is the key bacterial organelle required for motility, is linked to an extracellular hook and a flagellar filament (Minamino et al., 2008; Li and Sourjik, 2011).

ATPases, which are thought to provide the energy for the secretion process (Paul et al., 2008), are an essential and highly conserved component of all T3SSs. Thus, ATPases are essential for T3SS function and bacterial virulence. Several T3SS ATPases from animal and plant pathogenic bacteria have been identified and characterized, including EscN of *E. coli* (Andrade et al., 2007), InvC and SscN of *Salmonella* (Eichelberg et al., 1994; Akeda and Galan, 2004; Yoshida et al., 2014), YscN of *Yersinia* (Woestyn et al., 1994; Blaylock et al., 2006), and HrcN of *Xanthomonas* (Lorenz and Buttner, 2009), and they show significant amino acid sequence homology to the catalytic β subunit of F0F1 ATPases and are able to hydrolyze ATP (Zarivach et al., 2007). Previous evidence indicated that ATP hydrolysis by T3SS ATPases is required for the dissociation of effector proteins from their cognate chaperones. In addition, it has been shown that ATPases are involved in the unfolding of effector proteins before their secretion (Minamino and MacNab, 2000; Jouihri et al., 2003; Akeda and Galan, 2005; Pallen et al., 2006).

Most bacteria contain only one T3SS, but some pathogens harbor multiple T3SSs, which function independently in different aspects of pathogenesis (Moest and Meresse, 2013). Intestinal pathogenic *E. coli*, such as enteropathogenic *E. coli* (EPEC), and enterohemorrhagic *E. coli* (EHEC), use the locus of enterocyte effacement (LEE)-encoded T3SS to cause attaching/effacing lesions and diarrheal disease (Franzin and Sircili, 2015). A second T3SS, named *E. coli* type III secretion system 2 (ETT2), was identified by sequencing the genome of an EHEC O157 strain (Hayashi et al., 2001). The intact ETT2 cluster contains at least 35 open reading frames, 19 of which are homologous to those of *Salmonella* pathogenicity island 1 (Hansen-Wester and Hensel, 2001; Hayashi et al., 2001). ETT2 is known to be present, in whole or in part, in the majority of *E. coli* strains. It is most frequent in intestinal pathogenic *E. coli* strains, but of low prevalence in uropathogenic *E. coli* (UPEC) strains (Miyazaki et al., 2002; Prager et al., 2004; Ren et al., 2004; Cheng et al., 2012; Zhou et al., 2014). Our previous study demonstrated that ETT2 is more widely distributed in avian pathogenic *E. coli* (APEC) isolates and exhibits more ETT2 isoforms compared with human extraintestinal pathogenic *E. coli* (ExPEC), such as UPEC and newborn meningitis *E. coli* (NMEC; Wang et al., 2016). A sequence analysis indicated that there are gene deletions and premature stop codons in the ETT2 locus of most *E. coli* strains, which probably abolish the functions of ETT2. However, ETT2 still plays a role in regulating bacterial virulence (Zhang et al., 2004; Ideses et al., 2005; Sheikh et al., 2006; Yao et al., 2009). However, the role of ETT2 genes in the pathogenicity of APEC infection is less clear.

In the present study, we investigated the effects of the ETT2 ATPase EivC on the phenotype and virulence of APEC. Like other T3SS ATPases, EivC is homologous to the β subunit of F0F1 ATPases and it possesses ATPase activity. Our results verified that EivC is involved in motility, serum resistance, and intra-macrophage survival of APEC, suggesting that EivC is involved in the pathogenicity of APEC.

## Materials and Methods

### Bacterial Strains, Plasmids, and Growth Conditions

Strains and plasmids used in this study are listed in **Table 1**. APEC strain APCE94 was isolated from a chicken with clinical septicemia symptoms of colibacillosis in Jiangsu, China, and it can cause severe colibacillosis symptoms in chickens and ducks. This APEC strain is well-characterized; thus, it was used for infection studies, mutant construction, and functional assays (Wang et al., 2016). All *E. coli* strains were grown in Luria-Bertani (LB) medium at 37°C with aeration. When necessary, the LB medium was supplemented with ampicillin (Amp, 100 μg/mL) and chloramphenicol (Cm, 30 μg/mL), unless otherwise specified.

**Table 1:** Bacterial strains and plasmids used in this study.

Strains or plasmids	Characteristics	Reference
**Strains**		
APCE94	APEC wild-type strain, O78 serotype	This study
APCE94ΔeivC	*eivC* deletion mutant in APCE94	This study
APCE94CΔeivC	APCE94ΔeivC with plasmid pSTV28-eivC	This study
DH5α	F-, *Δ(lacZYA-argF)U169, recA1, endA1, hsdR17 (rk-, mk+), phoA, supE44, λ-*	TIANGEN
BL21 (DE3)	F-, *ompT, hsdS (r_B_^-^ m_B_^-^) gal, dcm* (DE3)	TIANGEN
**Plasmids**		
pET28a(+)	Kan, F1 origin, His tag	Novagen
pET28a-eivC	pET28a (+) carrying *eivC* gene	This study
pSTV28	Cm, lacZ	Takara
pSTV28-eivC	pSTV28 derivative harboring *eivC* gene	This study
pKD46	Amp; expresses λ red recombinase	Datsenko and Wanner, 2000
pKD3	Cm gene, template plasmid	Datsenko and Wanner, 2000
pCP20	Cm, Amp, yeast Flp recombinase gene, FLP	Datsenko and Wanner, 2000

### Expression and Purification of a Histidine-Tagged EivC Fusion Protein (His-EivC)

The open reading frame of APCE94 *eivC* was amplified and cloned into pET28a, yielding plasmid pET28a-eivC. The His-EivC fusion protein was expressed in *E. coli* BL21 (DE3) cells by induction with 1 mM isopropyl-β-D-thiogalactopyranoside. Protein purification was performed using a HisTrap high-performance column (GE Healthcare, Shanghai, China) according to the manufacturer’s guidelines. The final protein concentration was determined by the Bradford method using a SmartSpec 3000 spectrophotometer (Bio-Rad, Shanghai, China).

### ATPase Activity Assays

ATPase activity of the His-EivC fusion protein was determined using an ATPase/GTPase activity assay kit (Sigma, Shanghai, China) that measures the amount of inorganic phosphate (Pi) generated during hydrolysis, according to the manufacturer’s instructions. ATPase activity was expressed as 1 μmol phosphate released by 1 mg protein per min. All experiments were performed in triplicate.

### Construction of Mutant and Complementation Strains

An isogenic mutant strain APCE94ΔeivC was constructed based on the lambda Red recombinase system (Datsenko and Wanner, 2000). Briefly, the *eivC* gene was replaced with a chloramphenicol resistance cassette, which was polymerase chain reaction (PCR)-amplified from plasmid pKD3. Then, the chloramphenicol resistance cassette was cured by plasmid pCP20. The mutant strain APCE94ΔeivC was confirmed by PCR and sequencing. Promoter prediction analyses were conducted with prediction program tools available at http://www.fruitfly.org/seq_tools/promoter. A complementation strain was generated by cloning the *eivC* gene and its predicted promoter into plasmid pSTV28 using the primer pair eivCCo-F and eivCCo-R (**Table 2**). The resulting plasmid, pSTV28-eivC, was transformed into APCE94ΔeivC to generate the complementation strain APCE94CΔeivC.

**Table 2:** Primers used in this study.

Primers	Sequence (5′ to 3′)^a^	Target genes	Products size
eivCEx-F	CGCGGATCCATGTCTTGCGATATGGAGC	*eivC*	1317 bp
eivCEx-R	CCCAAGCTTCTTAACAATTCGTTCAAGCTCTT	*eivC*	
eivCMu-UF	AGAAGTTAACCCTACTGGACCC	Upstream region of *eivC*	767 bp
eivCMu-UR	GAAGCAGCTCCAGCCTACACTTGCCAGCTTGCATAAATTT	*eivC*	
eivCMu-CF	AAATTTATGCAAGCTGGCAAGTGTAGGCTGGAGCTGCTTC	pKD3	1014 bp
eivCMu-CR	CTGATAAGTCGATTAACTT CATATGAATATCCTCCTTAG	pKD3	
eivCMu-DF	CTAAGGAGGATATTCATATGAAGTTAATCGACTTATCAG	*eivC*	671 bp
eivCMu-DR	GGATTCCTGTCAACCTAGAGAG	Downstream region of *eivC*	
C1	TTATACGCAAGGCGACAAGG	pKD3	
C2	GATCTTCCGTCACAGGTAGG	pKD3	
eivCup-F	GGCTGATAGCAATTCGCC	Upstream region of *eivC*	3003 bp
eivCdown-R	CTGCGGTCCATTTATTTTGT	Downstream region of *eivC*	
eivCCo-F	GCGGAATTCGCCTTAGCATTATGGAGCCC	Upstream region of *eivC*	2031 bp
eivCCo-R	CGCGGATCCGCTGTTGAACTATTTCCTTGTTAA	Downstream region of *eivC*	
dnaE RT-F	ATGTCGGAGGCGTAAGGCT	*dnaE*	181 bp
dnaE RT-R	TCCAGGGCGTCAGTAAACAA	*dnaE*	
motA RT-F	GCTTTCTAATCTGAACGGTTACGC	*motA*	96 bp
motA RT-R	TCCAGCTCAATAAACGACGGAC	*motA*	
flgB RT-F	GCAGCAAACATCGCCAATGCC	*flgB*	101 bp
flgB RT-R	GTTGCATCCCGTCCACGTT	*flgB*	
flgD RT-F	GCACAAATCAGCACGGTCAGCG	*flgD*	122 bp
flgD RT-R	ATCATCACGCCGTGACCGATCA	*flgD*	
flgF RT-F	ACGTCGCGTTGCAGCAGGAT	*flgF*	128 bp
flgF RT-R	TATCACCGGATGCCCCTGAA	*flgF*	
fliC RT-F	CCAGTAGCTCCGTTAATCCCT	*fliC*	132 bp
fliC RT-R	AGTTTTGCTCAGTACACCGGAA	*fliC*	
fimC RT-F	GCCGATGGTGTAAAGGATGG	*fimC*	127 bp
fimC RT-R	AACTTTCCCGATCCTGTGGC	*fimC*	
ompA RT-F	GCTGAGCCTGGGTGTTTCCT	*ompA*	171 bp
ompA RT-R	TCCAGAGCAGCCTGACCTTC	*ompA*	
aatA RT-F	CCGTACCCGTGTCGCTGTTAC	*aatA*	98 bp
aatA RT-R	CAGCATTATCAGCATTGCCACT	*aatA*	
iss RT-F	CCGACAGCAGTAACACCAAAGG	*iss*	105 bp
iss RT-R	TTCTGCACCGCCAACAAATT	*iss*	
tsh RT-F	GCACGAACTGGGAAGTATGGA	*tsh*	118 bp
tsh RT-R	GGCATAGAAACCACCACCCC	*tsh*	
fyuA RT-F	TTGGCGACCAGGGTAAGAGC	*fyuA*	145 bp
fyuA RT-R	AGACCCGCAGTAGGCACGAT	*fyuA*	
iucD RT-F	GCTGGGTAGCAGACGGATAT	*iucD*	95 bp
iucD RT-R	GCATCACTGCCGATTCTTTA	*iucD*	
chβactin-F	GAGAAATTGTGCGTGACATCA	*β-actin*	152 bp
chβactin-R	CCTGAACCTCTCATTGCCA	*β-actin*	
chIL1-F	TGGGCATCAAGGGCTACA	*IL-1*	224 bp
chIL1-R	TCGGGTTGGTTGGTGATG	*IL-1*	
chIL8-F	TTGGAAGCCACTTCAGTCAGAC	*IL-8*	120 bp
chIL8-R	GGAGCAGGAGGAATTACCAGTT	*IL-8*	

*^a^Restriction sites are underlined.*

### Growth Curves and Motility Assays

To detect the effect of EivC on the growth rate of APEC, the growth kinetics of the APCE94, APCE94ΔeivC, and APCE94CΔeivC strains were determined in LB medium as described previously (Wang et al., 2014). Briefly, bacteria were incubated at 37°C with shaking, and the optical density of each strain was monitored at 1 h intervals by spectrophotometry.

A motility assay was performed as described previously (Han et al., 2015). Briefly, bacterial cultures were stabbed onto LB soft agar motility plates (0.5% agar) and incubated at 37°C. Bacterial motility haloes were measured after a 12 h incubation.

### Transmission Electron Microscopy (TEM)

Morphological changes of the APCE94, APCE94ΔeivC, and APCE94CΔeivC strains were determined by transmission electron microscopy (TEM). Briefly, each strain was grown on LB agar plates and suspended and washed with phosphate-buffered saline (PBS). Then, the bacterial pellets were negatively stained using 2% phosphotungstic acid (Sigma). Finally, the stained bacteria were deposited on a carbon-coated grid, followed by observation under a FEI T12 transmission electron microscope (FEI, Ltd, Hillsboro, OR, USA).

### Serum Bactericidal Assay

A bactericidal assay was performed in 96-well plates as described previously (Wang et al., 2014). Briefly, specific-pathogen-free (SPF) chicken sera were diluted to 12.5, 25, and 50% in PBS. Bacteria were incubated with different diluted sera or heat-inactivated sera (negative control) at 37°C for 30 min. Then, the bacteria were enumerated by plating on LB agar plates.

### Bacterial Adhesion and Invasion Assays

Bacterial adhesion and invasion assays were performed as described previously (Wang et al., 2011). Chicken embryo fibroblast DF-1 cell monolayers were washed with Dulbecco’s modified Eagle’s medium (DMEM) without fetal bovine serum, and infected with bacteria at a multiplicity of infection (MOI) of 100 for 2 h at 37°C under 5% CO_2_. After washing with PBS, the cells were lysed with 0.5% Triton X-100, and bacteria were counted by plating on LB agar plates. For invasion assays, bacterial infection of cell cultures was performed as described for the bacterial adhesion assays. After a 1 h incubation, cells were washed and treated subsequently with DMEM containing gentamicin (100 μg/mL) for 1 h to kill extracellular bacteria. Then, the monolayers were washed and lysed with 0.5% Triton X-100, and invasive bacteria were enumerated by plating on LB agar plates. DH5α or DF-1 cells only were used as controls in all experiments. Assays were performed three times in triplicate.

### Bacterial Intracellular Survival Assays

Bacterial intracellular survival was monitored as described previously (Wang et al., 2014). Briefly, avian HD-11 cells were infected with bacteria at a MOI of 100 for 1 h, as described for the invasion assays. Extracellular bacteria were killed with DMEM containing gentamicin (100 μg/mL) for 1 h, and intracellular bacteria were released, and they were defined as the initial number of invasive bacteria. Then, cells were grown in DMEM containing 10 μg/mL gentamicin for an additional 6, 12, or 24 h and lysed. Intracellular survival was expressed as the change (n-fold) in the bacterial number at a given time point relative to the initial number of invasive bacteria. This assay was performed three times in triplicate.

### Animal Infection Experiments

All animal experiments were conducted in strict accordance with the Guidelines on the Humane Treatment of Laboratory Animals (Ministry of Science and Technology of the People’s Republic of China, Policy No. 2006 398) and were approved by the Institutional Animal Care and Use Committee at the Shanghai Veterinary Research Institute (permit No: Shvri-Po-0243).

The virulence of the APCE94, APCE94ΔeivC, and APCE94CΔeivC strains to ducks was determined. APEC strains were grown to the exponential phase and collected, washed twice in PBS, and then adjusted to the appropriate doses. Groups of eight 7-day-old ducks were inoculated intra-tracheally with 10^8^ colony-forming units (CFUs) of bacteria, or with PBS as negative controls. Mortality was monitored daily until 7 days post-infection.

Bacterial colonization was determined during systemic infections, as described previously (Wang et al., 2011, 2014). Briefly, groups of eight 7-day-old ducks were infected intra-tracheally with a bacterial suspension containing 10^8^ CFUs. At 24 h post-infection, ducks were bled, euthanized, and dissected. Bacterial loads in the blood were counted by plating onto LB agar plates. The liver, spleen, and lung were collected, weighed, and homogenized. The homogenates were diluted and plated onto LB agar to determine the bacterial numbers.

### Quantitative Real-Time Reverse Transcription PCR (qRT-PCR)

Expression of bacterial virulence genes in the APCE94, APCE94ΔeivC, and APCE94CΔeivC strains was investigated by quantitative real-time reverse transcription PCR (qRT-PCR) as described previously (Wang et al., 2011). In addition, expression of interleukin (IL)-1β and IL-8 genes in HD-11 cells infected with the APCE94, APCE94ΔeivC, or APCE94CΔeivC strains was also investigated. In brief, total RNA was isolated from bacterial cultures or bacteria-infected HD-11 cells using TRIzol^®^ reagent (Invitrogen, Carlsbad, CA, USA). Contaminating DNA was removed from the samples with RNase-free DNase I (TaKaRa, Dalian, China). cDNA synthesis was performed using the PrimeScript^®^ RT reagent kit (TaKaRa) according to the manufacturer’s protocol. qRT-PCR was performed using SYBR^®^ *Premix Ex* Taq^TM^ (TaKaRa) and gene-specific primers (**Table 2**). Relative gene expression was normalized to the expression of the housekeeping gene *dnaE* or β-actin via the ΔΔCT method. The PCR efficiency (>90%) for each of the genes was verified via standard dilution curves. The assay was performed in duplicate and repeated three times.

### Statistical Analyses

Statistical analyses were conducted using the GraphPad Software package (GraphPad Software, La Jolla, CA, USA). One-way analysis of variance (ANOVA) was used to analyze motility and invasion assay data, and two-way ANOVA was performed to analyze the survival assays and qRT-PCR results. The animal infection data were analyzed using the non-parametric Mann–Whitney *U*-test. The mean values are shown in the figures. Statistical significance was established at *p* < 0.05.

## Results

### Sequence Analysis of the ETT2 ATPase EivC in APCE94

**FIGURE 1:**
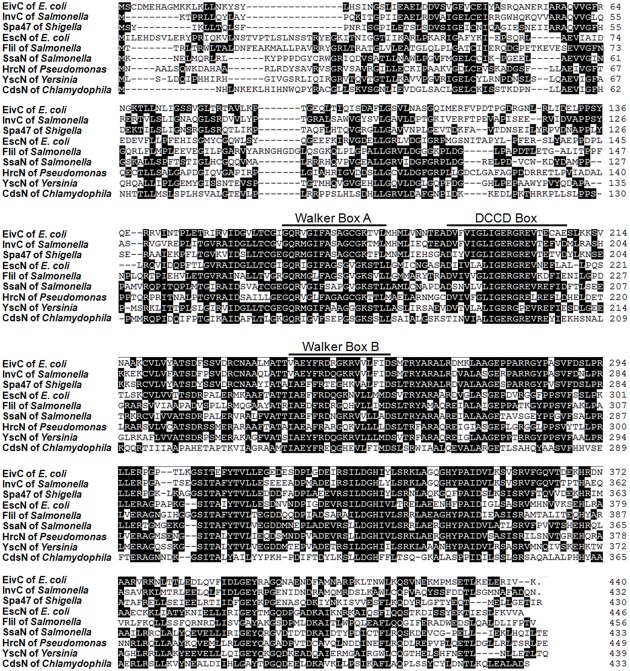
**Sequence analysis of ETT2 and EivC.** Amino acid sequence comparisons of EivC with other T3SS ATPases. The locations of predicted Walker boxes A and B and a dicyclohexylcarbodiimide-binding site (DCCD box) within the amino acid sequence of EivC are indicated.

Our previous study showed that APCE94 harbors a type B ETT2 cluster. There is a 4.99-kb gene deletion in the *eiv* operon, which truncates the *eivA* and *eivF* genes (Wang et al., 2016). An amino acid sequence analysis indicated that EivC contains a glycine-rich region (Walker box A) and a nucleotide-binding protein region (Walker box B), which was the best conserved motif of the F0F1 ATPase (Zarivach et al., 2007). Moreover, a dicyclohexylcarbodiimide (DCCD) box was also present around a conserved glutamic acid residue, which reacted with DCCD in other ATP-binding proteins (**Figure 1**). These data suggest that the *eivC* gene likely encodes a F0F1 ATPase.

### ATPase Activity of EivC

**FIGURE 2:**
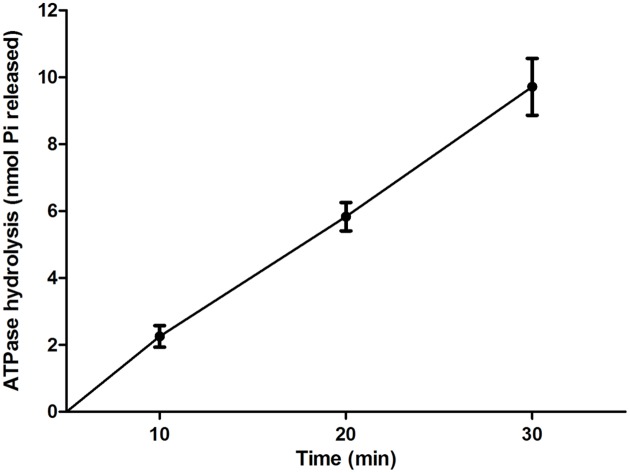
**ATPase activity of EivC.** The His-EivC fusion protein was purified by nickel-affinity chromatography. ATP hydrolysis by the purified His-EivC fusion protein was determined using a malachite green assay over a period of 30 min. Error bars indicate standard deviations.

To determine whether EivC possess ATPase activity, a His-EivC fusion protein was purified and tested for its ability to hydrolyze ATP. The purified His-EivC fusion protein showed ATPase activity, with phosphate release rate of 0.28 ± 0.08 μmol/min/mg (**Figure 2**). This activity is comparable to that of the homologous ATPase InvC of *Salmonella enterica* serovar *Typhimurium* (Eichelberg et al., 1994; Akeda and Galan, 2004).

### Inactivation of EivC Reduces the Motility of APCE94

The *eivC* mutant and complementation strains were successfully constructed using the lambda Red recombination system and plasmid pSTV28. No significant differences were observed in the generation times among the APCE94, APCE94ΔeivC, and APCE94CΔeivC strains during growth in LB medium (data not shown). After curing the chloramphenicol resistance cassette from the *eivC* gene, essential ribosomal binding sequences for the translation of downstream genes remained. Therefore, deletion of the *eivC* gene did not have a polar effect on the expression of downstream genes in ETT2 (data not shown).

**FIGURE 3:**
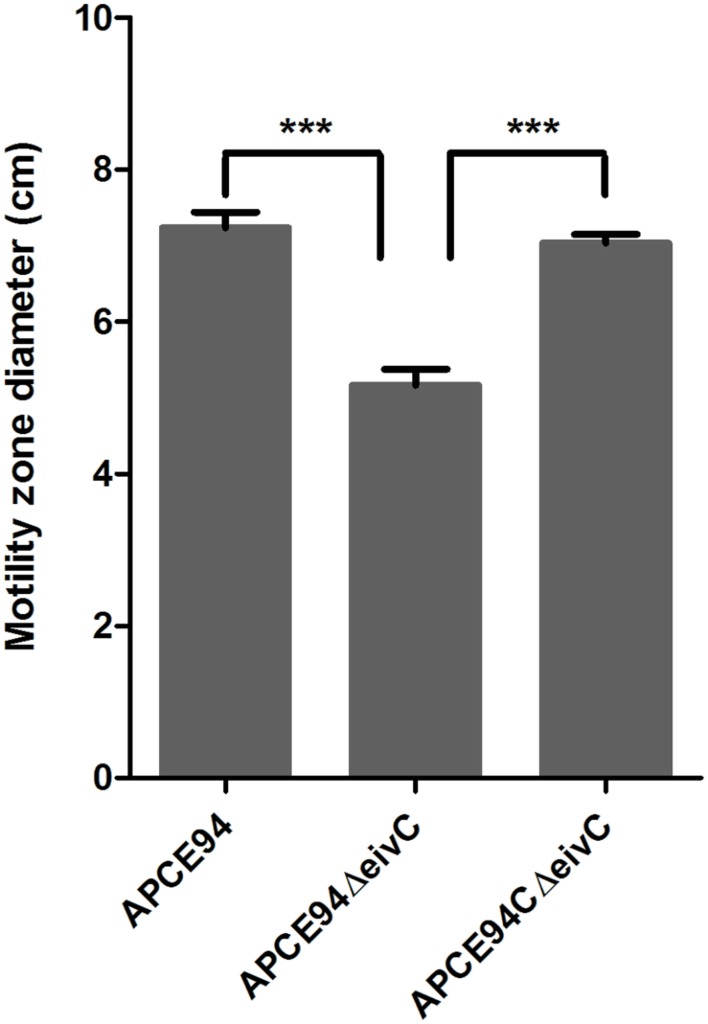
**Effects of EivC on bacterial motility.** Mutant strain APCE94ΔeivC exhibited a smaller motility diameter than the wild-type strain APCE94 and the complementation strain APCE94CΔeivC. Bars represent the average motility diameters of triplicate motility plates. Error bars represent the standard deviation. One-way ANOVA was used for the motility analysis (^∗∗∗^*p* < 0.001).

The F0F1 ATPase is similar to the FliI ATPase, which is an essential component of the bacterial flagellar motor that is required for bacterial motility (Minamino et al., 2008). Thus, we incubated the APCE94, APCE94ΔeivC, and APCE94CΔeivC strains on semisolid LB agar plates to examine whether EivC contributes to bacterial motility. The results showed that the diameter of the halo of the APCE94ΔeivC strain was significantly smaller than that of the APCE94 strain (*p* < 0.001), whereas the diameter of the halo of the APCE94CΔeivC strain was almost equal to that of the APCE94 strain (**Figure 3**). Therefore, deletion of *eivC* reduced the motility of APCE94.

### Knockout of *eivC* Leads to Fewer Flagella and Augmented Fimbriae

**FIGURE 4:**
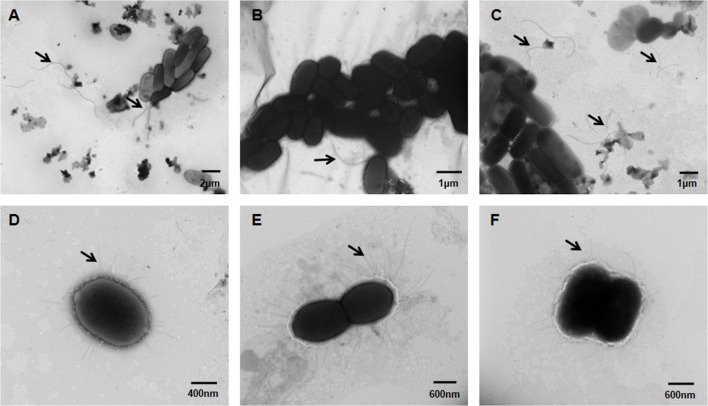
**Transmission electron micrographs of APEC strains.** Bacteria were grown overnight on LB agar, and then harvested, fixed, and negatively stained with 2% phosphotungstic acid. Numerous long flagella were observed on the surfaces of the wild-type strain APCE94 **(A)** and the complementation strain APCE94CΔeivC **(C)**. Few flagella were observed on the mutant strain APCE94ΔeivC **(B)**. Augmented fimbriae surrounding the bacteria were observed on the mutant strain APCE94ΔeivC **(E)**, compared with those of the wild-type strain APCE94 **(D)** and the complementation strain APCE94CΔeivC **(F)**.

The influence of *eivC* on the bacterial morphological structure was examined by TEM. The results revealed remarkable differences in the flagella and fimbriae between the APCE94 and APCE94ΔeivC strains. The APCE94ΔeivC strain produced fewer flagella (including attached and broken flagella) than the APCE94 and APCE94CΔeivC strains (**Figures 4A–C**). In contrast, numerous long fimbriae surrounding the bacteria were observed in the APCE94ΔeivC strain, compared with that of the APCE94 strain. The fimbrial structures of the APCE94CΔeivC strain were similar to those of the APCE94 strain (**Figures 4D–F**). According to these results, deletion of *eivC* leads to impaired flagella production and augmented fimbriae on the bacterial surface.

### EivC Is Required for the Serum Resistance of APCE94

**FIGURE 5:**
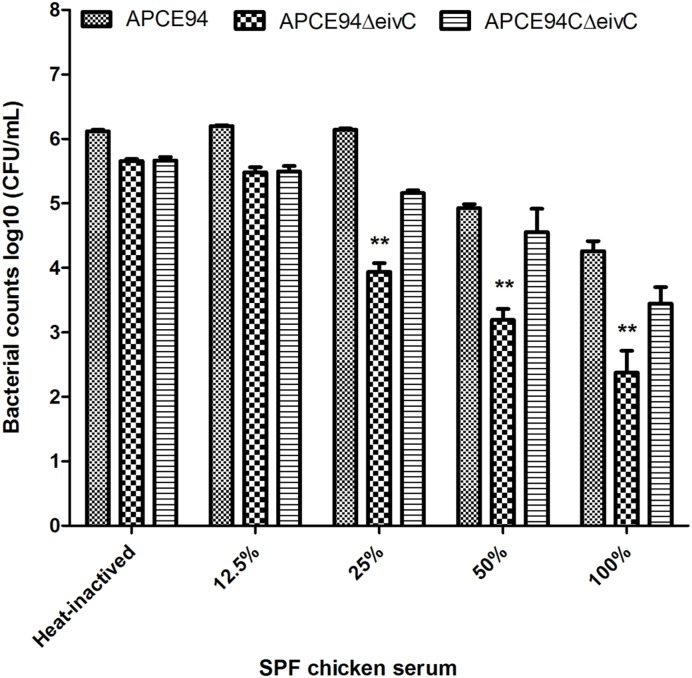
**Bacterial resistance to SPF chicken serum.** The APCE94, APCE94ΔeivC, and APCE94CΔeivC strains were incubated with SPF chicken sera at different dilutions at 37°C for 30 min, and the surviving bacteria were enumerated. This data revealed that the mutant strain APCE94ΔeivC was significantly and highly susceptible to SPF chicken sera. A two-way ANOVA was performed for the survival assays (^∗∗^*p* < 0.01).

During colonization and survival in a host, resistance to serum killing is necessary for APEC pathogenicity (La Ragione and Woodward, 2002; Mellata et al., 2003). The serum bactericidal assay showed that the survival of the APCE94ΔeivC strain in SPF chicken serum was significantly reduced compared with that of the APCE94 strain (*p* < 0.01). Furthermore, the resistance to serum killing was restored in the APCE94CΔeivC strain (**Figure 5**). These observations indicate that EivC is required for the serum resistance of APCE94.

### EivC Facilitates the Intracellular Survival of APCE94 in Avian HD-11 Cells

To investigate whether *eivC* affects bacterial adhesion and invasion capacities, the avian cell line DF-1 was infected with the APCE94, APCE94ΔeivC, or APCE94CΔeivC strains. Although the APCE94ΔeivC strain showed increased adherence to, and invasion of, DF-1 cells, these values were not significantly different from those of the APCE94 strain (data not shown). These results suggest that EivC does not affect the capacity of APEC to adhere to and invade into DF-1 cells.

**FIGURE 6:**
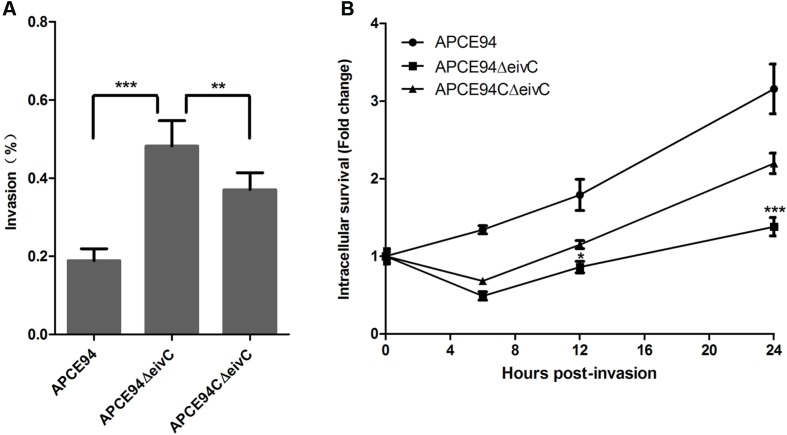
**Bacterial invasion and intracellular survival assays. (A)** Invasion assay with avian HD-11 cells. The mutant strain APCE94ΔeivC showed significantly increased invasion to HD-11 cells, compared with the wild-type strain APCE94 and the complementation strain APCE94CΔeivC. Values are averages of three independent experiments. Error bars indicate standard deviations. Statistical significance was assessed using one-way ANOVA (^∗∗^*p* < 0.01; ^∗∗∗^*p* < 0.001). **(B)** Intracellular survival assay. Inactivation of EivC decreased its intracellular survival within avian HD-11 cells compared with APCE94, which was expressed as the fold change in bacterial numbers at 6, 12, and 24 h relative to the number of initial invasive bacteria. A two-way ANOVA was performed for the survival assays (^∗^*p* < 0.05; ^∗∗∗^*p* < 0.001).

To determine whether *eivC* influences the intracellular survival and replication of APEC, the intracellular survival of the APCE94 strains in HD-11 cells was assessed at 0, 6, 12, and 24 h post-invasion. Unlike the case of DF-1 cells, the APCE94ΔeivC strain exhibited significantly increased invasiveness of avian HD-11 cells, compared with the APCE94 strains (*p* < 0.001). However, the intracellular survival of the APCE94ΔeivC strain in avian HD-11 cells was significantly lower than that of the APCE94 strain at all time points tested (*p* < 0.05). Complementation with *eivC* partially restored the invasion and intracellular survival capacities (**Figure 6**). These findings indicate that EivC contributes to the intracellular survival of APEC in chicken macrophages.

### EivC Contributes to Bacterial Colonization and Virulence during Systemic Infection *In vivo*

**FIGURE 7:**
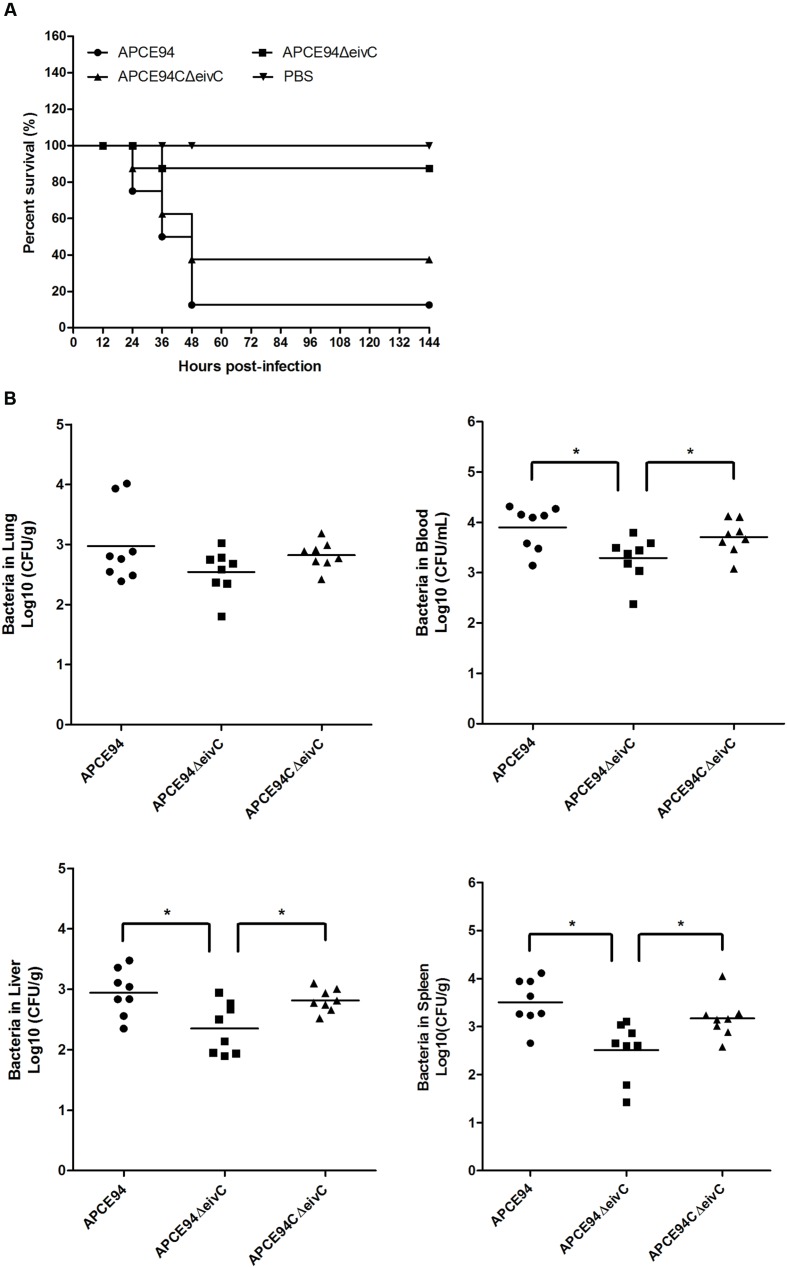
**Animal systemic infection experiments. (A)** Determination of bacterial virulence. Seven-day-old ducks were inoculated intra-tracheally with the APCE94, APCE94ΔeivC, or APCE94CΔeivC strains at 10^8^ colony-forming units (CFUs). Ducks injected with PBS were used as negative controls. Survival was monitored until 7 days post-infection. **(B)** Bacterial colonization and proliferation in ducks. Groups of eight 7-day-old ducks were intra-tracheally infected with 10^8^ CFUs of bacteria. Bacteria were recovered from the lung, blood, liver and spleen at 24 h post-infection. A non-parametric Mann–Whitney *U*-test was conducted to determine statistical significance (^∗^*p* < 0.05).

To investigate the effect of EivC on bacterial pathogenicity, the virulence of the APCE94, APCE94ΔeivC, and APCE94CΔeivC strains was compared using the duck model. As shown in **Figure 7A**, the mortality rate following infection with the APCE94ΔeivC strain (12.5%, 1/8) was significantly lower than that after infection with the APCE94 strain (87.5%, 7/8). The mortality rate following infection with the APCE94CΔeivC strain was restored to 62.5% (5/8). These results provide evidence that EivC contributes to the virulence of APEC.

To measure the influence of *eivC* on APEC colonization *in vivo*, a systemic infection experiment was performed to assess bacterial proliferation in ducks. The bacterial loads in the lung, blood, liver, and spleen were determined at 24 h post-infection. The results showed a significant reduction in the number of recovered APCE94ΔeivC bacteria in the blood, liver, and spleen, compared with that of the APCE94 strain (*p* < 0.05), while the APCE94CΔeivC strain exhibited a higher bacterial colonization capacity. There were no significant differences in the colonization of the lungs among the APCE94ΔeivC, APCE94, and APCE94CΔeivC strains (*p* > 0.05; **Figure 7B**). These results indicate that EivC is involved in colonization and survival during infection *in vivo*.

### Expression Profiling of Flagellar, Fimbrial, and Virulence Genes

**FIGURE 8:**
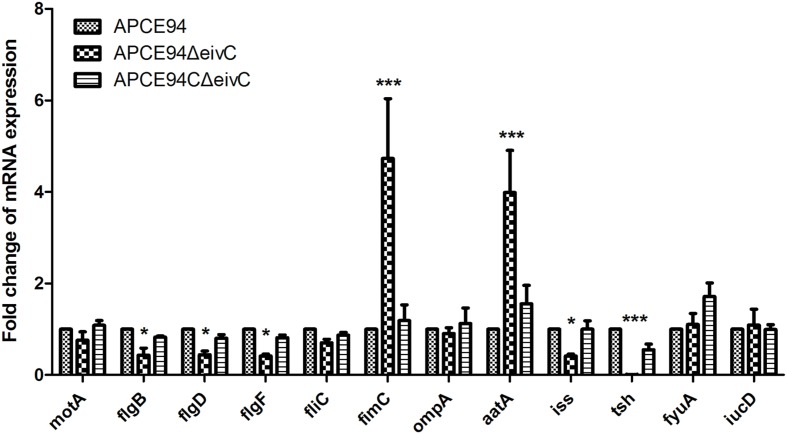
**Quantification of virulence gene expressions.** Quantitative real-time PCR (qRT-PCR) was performed to measure the expression levels of the flagellar genes *motA*, *flgB, flgD*, *flgF*, and *fliC*, the type 1 fimbrial gene *fimC*, and the virulence genes *ompA*, *aatA*, *iss*, *tsh*, *fyuA*, *iucD* in the APCE94, APCE94ΔeivC, and APCE94CΔeivC strains. Data were normalized to the housekeeping gene *dnaE*. The results are shown as relative expression ratios compared to expression in the wild-type strain APCE94. Statistical significance was assessed using two-way ANOVA (^∗^*p* < 0.05; ^∗∗∗^*p* < 0.001).

To further explore the effect of *eivC* on the phenotypes and virulence of APEC, the expression of fimbrial, flagellar, and virulence genes in the APCE94, APCE94ΔeivC, and APCE94CΔeivC strains was analyzed by qRT-PCR. The transcription levels of the flagellar genes *motA*, *flgB*, *flgD*, *flgF*, and *fliC*, the type 1 fimbriae gene *fimC*, and the virulence genes *ompA*, *aatA*, *iss*, *tsh*, *iucD*, *fyuA*, and *iucD* in the APCE94ΔeivC strain were quantified as the fold-change relative to those of the APCE94 strain. As shown in **Figure 8**, the mRNA levels of the *flgB*, *flgD*, *flgF*, *iss*, and *tsh* genes were significantly downregulated in the APCE94ΔeivC compared with those in the APCE94 strain (*p* < 0.05; *p* < 0.001). By contrast, *fimC* and *aatA* expression was significantly upregulated in the APCE94ΔeivC strain (*p* < 0.001). The expression levels of these genes were restored in the APCE94CΔeivC strain. The qRT-PCR results are consistent with the phenotypes and virulence levels observed in this study.

### Determination of Cytokine Expression in APEC-Infected Avian HD-11 Cells

**FIGURE 9:**
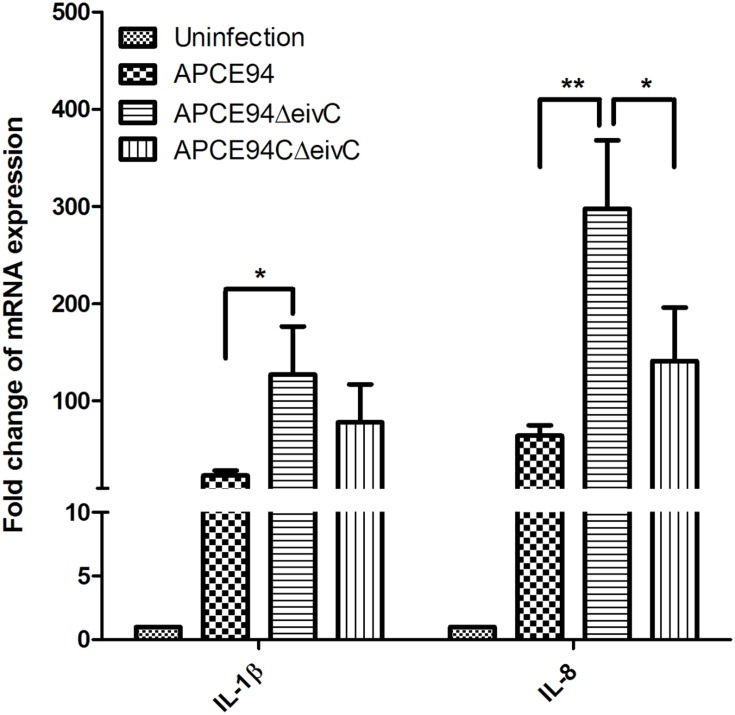
**Cytokine expressions in APEC-infected avian HD-11 cells.** Avian HD-11 cells were infected with the APCE94, APCE94ΔeivC, or APCE94CΔeivC strains. The mRNA levels of IL-1β and IL-8 in bacteria-infected HD-11 cells were analyzed at 3 h post-invasion by qRT-PCR. Data were normalized to the β-actin gene. Samples were calibrated to gene expression in uninfected HD-11 cells. Expression of IL-1β and IL-8 was significantly upregulated in cells infected with the APCE94ΔeivC strain, compared with those infected with the APCE94 strain. Statistical significance was assessed by two-way ANOVA (^∗^*p <* 0.05; ^∗∗^*p <* 0.01).

It is known that pathogen infection can stimulate the host inflammatory response (Raymond et al., 2013). Thus, the expression of the inflammatory cytokines IL-1β and IL-8 in APEC-infected avian HD-11 cells was assessed at 3 h post-invasion by qRT-PCR. The results showed that the expression of IL-1β and IL-8 was significantly upregulated in APEC-infected cells, compared with those in uninfected cells. Moreover, the IL-1β and IL-8 expression levels were significantly increased in HD-11 cells infected the APCE94ΔeivC strain, compared with those in HD-11 cells infected with the APCE94 strain (*p* < 0.05 and *p* < 0.01, respectively). The expression levels of IL-1β and IL-8 were partially restored in the APCE94CΔeivC strain (**Figure 9**).

## Discussion

Pathogenic *E. coli* are remarkably diverse, and they cause a wide range of diseases, ranging from gastroenteritis and diarrhea to extraintestinal infections, such as neonatal meningitis, urinary tract infections, pneumonia, and septicemia. Although the virulence factors and pathogenic mechanisms vary, pathogenic *E. coli* share some virulence strategies, such as a T3SS. ETT2 is known to be present, in whole or in part, in the majority of *E. coli* strains. Our previous study verified that the prevalence of ETT2 in APEC, similar to intestinal pathogenic *E. coli*, was markedly higher than that in human ExPEC (UPEC and NMEC); thus, it might be a potential risk to human health (Wang et al., 2016).

ATPases have been thought to be the key energizers of T3SSs, and their roles have been studied intensively (Paul et al., 2008). In this study, we determined the effects of the putative ETT2 ATPase EivC on the phenotype and virulence of APEC. Similar to other T3SS ATPases, EivC is homologous to the β subunit of F0F1 ATPases and contains the conserved Walker boxes A and B. We demonstrated that EivC possesses ATPase activity, hydrolyzing ATP at a rate of 0.28 ± 0.08 μmol/min/mg in this study. This ATP hydrolysis rate is similar to those of other T3SS ATPase family members, such as InvC (Eichelberg et al., 1994; Akeda and Galan, 2004), EscN (Andrade et al., 2007), SsaN (Yoshida et al., 2014), FliI (Fan and Macnab, 1996), YsaN (Chatterjee et al., 2013), HrcN (Lorenz and Buttner, 2009), and CdsN (Stone et al., 2008). Therefore, the functions of EivC in motility, serum resistance, and virulence *in vitro* and *in vivo* were evaluated.

T3SS ATPases are responsible for the export of flagellar- and virulence-related effector proteins (Cornelis and Van Gijsegem, 2000; Mota and Cornelis, 2005; Diepold and Wagner, 2014). Flagella are organelles whose main function is cell motility. Our results showed that inactivation of *eivC* decreased the production of flagella on the bacterial surface, thus reducing bacterial motility. However, type 1 fimbriae were augmented on the bacterial surface of the APCE94ΔeivC strain. Similarly, previous evidence indicated that inactivation of ETT2 affects the bacterial surface properties of septicemic *E. coli* (Ideses et al., 2005). The qRT-PCR analysis revealed that the APCE94ΔeivC strain exhibited downregulated flagellar gene (*flgB*, *flgD*, *flgF*) expression and upregulated fimbriae gene (*fimC*) expression. Moreover, previous evidence indicated that constitutive expression of type 1 fimbriae dramatically reduced the motility and flagellin expression of UPEC and *E. coli* K-12 MG1655 (Lane et al., 2007). Therefore, the increased fimbria expression in the APCE94ΔeivC strain might suppress flagellar gene expression, resulting in diminished motility.

T3SS ATPases are virulence factors, playing crucial roles in the pathogenicity of different pathogenic bacteria (Eichelberg et al., 1994; Fan and Macnab, 1996; Akeda and Galan, 2004; Blaylock et al., 2006; Andrade et al., 2007; Stone et al., 2008; Chatterjee et al., 2013). Here, we demonstrated that the ETT2 ATPase EivC is essential for APEC virulence. Inactivation of ATPase EivC led to significantly attenuated virulence, including impaired serum resistance, reduced intracellular survival in HD-11 cells, and diminished colonization in ducks. These defects were partially restored by complementation with *eivC*. Colonization and invasion play important roles during APEC infection. Fimbriae are known to contribute to adhesion to, and invasion of, host cells by bacteria (Luthje and Brauner, 2014). Previous studies showed that mutations of virulence genes (including *ibeA*, as well as those encoding the type VI secretion system, and the BarA-UvrY two component system) lead to a defect in expression of type 1 fimbriae, which may account for the diminished adhesion, invasion, and virulence of APEC (Herren et al., 2006; de Pace et al., 2010; Wang et al., 2011). Similarly, our study indicated that deletion of *eivC* increased the number and expression of type 1 fimbriae. It might be responsible for the increased invasiveness of HD-11 cells by the APCE94ΔeivC strain. However, the adherence to, and invasion of, DF-1 cells, as well as colonization of the lungs, did not differ significantly among the wild-type, mutant, and complementation strains. As well-known, in addition to colonization and invasion capacities, a number of virulence factors are also involved in the progress of infection, and these factors might be responsible for this phenomenon.

Avian pathogenic *Escherichia coli* initially infects poultry via the respiratory tract, and then it spreads systemically throughout the whole body. Thus, survival in serum is an important virulence parameter for APEC infection. There is a correlation between resistance to serum bactericidal effects and the capacity of APEC strains to cause septicemia and mortality (La Ragione and Woodward, 2002; Mellata et al., 2003). In addition, the capacity to survive within macrophages prevents the elimination of APEC by the host immune response. Bacterial surface components, such as lipopolysaccharide (LPS), capsules, as well as increased production of serum survival proteins, play important roles in resistance to serum and other factors present in macrophages. The bacteria surface structural alteration of the APCE94ΔeivC strain may contribute to reduced fitness within serum or a host. To determine whether the LPS of the APCE94ΔeivC strain was defective, LPS was purified from the APEC strains and subjected to sodium dodecyl sulfate-polyacrylamide gel electrophoresis and silver staining. However, no changes of the LPS profile were found among the mutant strain APCE94ΔeivC, the wild-type strain APCE94, and the complementation strain APCE94CΔeivC. However, the qRT-PCR results demonstrated that the transcriptional levels of virulence genes involved in serum resistance (*iss*; Foley et al., 2000), and a gene encoding an autotransporter protein (*tsh*; Dozois et al., 2000), were significantly decreased in the APCE94ΔeivC strain, which might be a reason for the serum resistance defects, as well as the reduced colonization and proliferation capacities and virulence *in vivo*. Indeed, it has been discovered that ETT2 can affect the expression of virulence genes outside the ETT2 cluster, and it can indirectly regulate the virulence of intestinal pathogenic *E. coli* (Zhang et al., 2004; Sheikh et al., 2006). Similarly, previous evidence has shown that ETT2, although degenerate, is involved in serum survival, invasion, and intracellular survival, which are necessary for the full virulence of septicemic *E. coli* and NMEC (Ideses et al., 2005; Yao et al., 2009). However, the underlying mechanisms are unclear.

Bacteria utilize T3SSs to deliver effector proteins into host cells, which play various roles in invasion, intracellular survival, and subversion of innate immune responses. During infection, bacteria and their components can induce strong inflammatory and immune responses. Nevertheless, pathogenic bacteria are able to efficiently modulate host inflammatory and immune responses, thereby facilitating their proliferation and infections (Figueira and Holden, 2012; Raymond et al., 2013). Pathogens utilize multiple different mechanisms to disrupt the inflammatory response, including nuclear factor kappa-light-chain-enhancer of activated B cells (NF-κB) and mitogen-activated protein kinase (MAPK) signaling pathways. In EHEC and EPEC, the LEE effector proteins NleE, NleB, NleC, NleH, and Tir cooperatively interact with different proteins of the NF-κB signaling pathway, and subsequently, prevent the production of the inflammatory cytokine IL-8 (Nadler et al., 2010; Vossenkamper et al., 2010; Yen et al., 2010; Ruchaud-Sparagano et al., 2011; Pham et al., 2012; Gao et al., 2013). In addition, the MAPK signaling pathway is targeted by the effectors Tir and NleD to inhibit the inflammatory response and cytokine production (Baruch et al., 2011; Sham et al., 2011). A number of T3SS effector proteins, which also play roles in the inhibition of the inflammatory response, have been identified in *Salmonella*, *Shigella*, and *Yersinia* (Haraga and Miller, 2003; Zurawski et al., 2009; Paquette et al., 2012; Sanada et al., 2012), whereas the degenerate ETT2 is thought to be a non-functional secretion system because no effector proteins were identified in laboratory conditions following treatment with known inducers of T3SSs in other bacteria (Ren et al., 2004). T3SSs are generally activated by interactions with host cells, which might be a reason for the failure to identify effector proteins. Our results showed that the ETT2 ATPase EivC suppressed the expression of the inflammatory cytokines IL-1β and IL-8, and facilitated bacterial infection and virulence. APEC might utilize unidentified ETT2 effector proteins or other bacterial surface components to interfere with the host inflammatory response. However, the exact mechanisms by which the ETT2 interferes with the inflammatory response remain unknown. Such mechanisms need to be investigated in the future to help us prevent poultry colibacillosis and potential human infections (Kline et al., 2012; Marshall and Finlay, 2014).

In summary, we characterized the functions of the putative ETT2 ATPase EivC in an APEC strain. EivC is homologous to the β subunit of F0F1 ATPases and it possesses ATPase activity. EivC was crucial for bacterial flagellation and motility. In addition, ATPase EivC was involved in serum resistance, intra-macrophage survival, proliferation *in vivo*, and subversion of host inflammatory response.

## Author Contributions

SW planned the experiments. SW, XL, XX, DY, DW, and YS conducted the experiments. SW, XL, XH, MT, CD, DP, and SY analyzed and discussed the experimental results. SW and SY wrote the manuscript.

## Conflict of Interest Statement

The authors declare that the research was conducted in the absence of any commercial or financial relationships that could be construed as a potential conflict of interest.
